# Metabolic reprogramming of macrophages during mycobacterial infection: a review of immunometabolic crosstalk and pathogen manipulation

**DOI:** 10.3389/fcimb.2026.1806805

**Published:** 2026-06-24

**Authors:** Xiaohe Lu, Yanping Zhao, Pingfan Qin, Yongquan Shi, Xiong Xiao

**Affiliations:** 1 Department of Laboratory Medicine, Affiliated Hospital of Shandong University of Traditional Chinese Medicine, Jinan, China; 2 Department of Clinical Laboratory Center, Shandong Second Provincial General Hospital, Jinan, China; 3 Department of Thyroid and Breast Surgery, Wuhan Union Hospital, Tongji Medical College, Huazhong University of Science and Technology, Wuhan, China

**Keywords:** amino acid metabolism, fatty acid metabolism, glycolysis, immunometabolic interplay, macrophages, mycobacterial infection, TCA cycle

## Abstract

Macrophages undergo dynamic metabolic reprogramming that critically shapes their functional polarization and antimicrobial responses during mycobacterial infection. This review integrates current knowledge on how infection reprograms major metabolic pathways in macrophages. Mycobacterial infection triggers a complex and often dual-purposed rewiring of glycolysis, the tricarboxylic acid (TCA) cycle, and amino acid metabolism. Pathogens actively manipulate these pathways to simultaneously suppress host antimicrobial effector functions and acquire nutrients for their own survival. Enhanced glycolysis, typically linked to M1 macrophages, can be exploited by the pathogen. Reprogramming of the TCA cycle, particularly through metabolites like itaconate, drives macrophages polarization toward an M2 phenotype that favors bacterial persistence. Amino acid metabolism becomes a site of metabolic competition where the bacterium secures substrates such as arginine and tryptophan to induce M2 phenotype, while the host attempts to sustain M1 macrophage functions through glutamine metabolism and the arginine nitric oxide pathway. Fatty acid metabolism further contributes to macrophage polarization in a context dependent manner. Understanding this immunometabolic interplay provides novel insights into tuberculosis pathogenesis and highlights metabolic pathways as potential targets for host-directed therapies. Future research should clarify the heterogeneity of metabolic responses across different mycobacterial species, infection stages, and macrophage subsets to guide therapeutic strategies.

## Introduction

Immunometabolism encompasses the concept that the metabolic state of immune cells determines their functional capabilities. As a vital component of the innate immune system, macrophages possess a range of critical functions, including phagocytosis, antigen presentation, immune regulation, and cytokine secretion. The functionality of macrophages is modulated by polarization signals, and their polarized states are closely associated with changes in metabolic intermediates. The metabolic pathways that predominantly influence the immune status of macrophages include glycolysis, the TCA cycle, fatty acid metabolism, and amino acid metabolism. Extensive research has demonstrated that macrophage metabolism is intricately linked to the pathogenesis and progression of various diseases, including inflammation, cancer, obesity, diabetes, and metabolic syndrome (Hotamisligil, 2017; Schwartz et al., 2017; Makowski et al., 2020).

Mycobacterial infection has afflicted humans for millennia, with evidence of *M. tuberculosis* infection dating back to approximately 3000 BCE (Zink et al., 2001). *M. tuberculosis*, the causative agent of tuberculosis (TB), is the most clinically significant pathogen among mycobacterial infection. Although macrophages play a crucial role in combating *M. tuberculosis* infections, they paradoxically serve as the primary target cells for these pathogens. *M. tuberculosis* infection induces alterations in macrophage polarization, which are closely associated with disease pathogenesis and progression. Specifically, *M. tuberculosis* infection promotes macrophage polarization toward the M1 phenotype, facilitating granuloma formation and enhancing bactericidal activity (Shim et al., 2020a). Recent studies have demonstrated that *M. tuberculosis* infections alter macrophage metabolic pathways, thereby affecting their immune functions. This review consolidates current knowledge on the impact of *M. tuberculosis* infections on macrophage glycolysis, the TCA cycle, amino acid metabolism, and fatty acid metabolism. It further explores the relationship between metabolic reprogramming and the resulting changes in immune function in infected macrophages.

## Distinct metabolic characteristics of M1 and M2 macrophages

**Figure 1:**
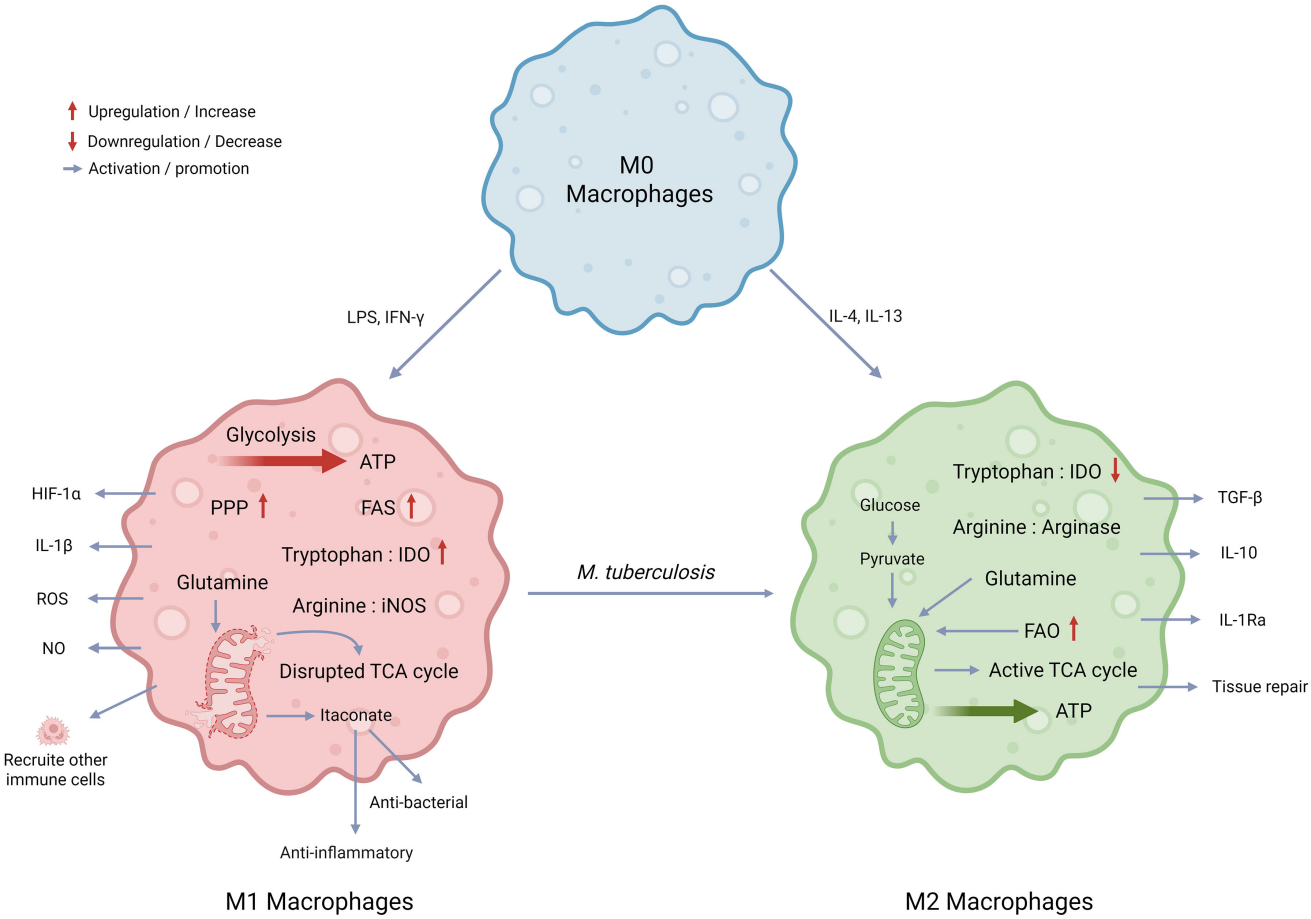
Metabolic reprogramming of M1 and M2 macrophages during *M. tuberculosis* infection. M0 macrophages differentiate into either classically activated M1 macrophages or alternatively activated M2 macrophages in response to LPS and IFN-γ, which drive M1 polarization, characterized by enhanced glycolysis, activation of the PPP, FAS, and disruption of the TCA cycle. M1 macrophages also exhibit altered amino acid metabolism, including increased iNOS-mediated arginine metabolism and elevated IDO-dependent tryptophan catabolism, leading to the production of NO, ROS, IL-1β, and HIF-1α, thereby promoting pro-inflammatory and antimicrobial responses. In contrast, IL-4 and IL-13 induce M2 polarization, which is associated with enhanced FAO, active TCA cycle, and increased ATP production. M2 macrophages preferentially utilize arginase-mediated arginine metabolism and exhibit altered glutamine and tryptophan metabolism, contributing to the secretion of anti-inflammatory mediators, including TGF-β, IL-10, and IL-1Ra, thereby facilitating immune regulation and tissue repair.

Macrophages exhibit considerable heterogeneity in both morphology and function, with the major subtypes being classically activated M1 macrophages and alternatively activated M2 macrophages (**Figure 1**). Upon exposure to bacterial lipopolysaccharide (LPS) and cytokines such as interferon-gamma (IFN-γ) secreted by type 1 T helper cells, unpolarized macrophages (M0 macrophages) differentiate into M1 macrophages (Martinez et al., 2008; Hobson-Gutierrez and Carmona-Fontaine, 2018). This differentiation process triggers the expression and secretion of inflammatory mediators, including interleukin-1β (IL-1β), hypoxia-inducible factor 1-α (HIF-1α), nitric oxide (NO), and reactive oxygen species (ROS), and the resulting M1 macrophages recruit other immune cells to eradicate pathogens (Lachmandas et al., 2016; Williams and O’Neill, 2018; Osada-Oka et al., 2019; Wang S. et al., 2019). The metabolic signature of M1 macrophages is characterized by increased glycolysis and a disrupted TCA cycle, with adenosine triphosphate (ATP) production predominantly relying on glycolysis (Galván-Peña and O’Neill, 2014; Williams and O’Neill, 2018) (**Figure 1**). Although glycolysis is less efficient in ATP generation, it enables macrophages to rapidly respond to the initial phase of inflammation and provide the necessary ATP for the synthesis of inflammatory mediators (Galván-Peña and O’Neill, 2014; Williams and O’Neill, 2018). Furthermore, reprogramming of the TCA cycle leads to the production of itaconate, a metabolite with both anti-bacterial and anti-inflammatory properties (Jo et al., 2019; O’Carroll et al., 2025). In contrast, stimulation with type 2 T helper cell cytokines such as interleukin-4 and interleukin-13 can induce the differentiation of M0 macrophages into M2 macrophages (Martinez et al., 2008; Hobson-Gutierrez and Carmona-Fontaine, 2018). M2 macrophages are characterized by an active TCA cycle and the expression and secretion of anti-inflammatory cytokines, including interleukin-10 (IL-10), transforming growth factor-β (TGF-β), and interleukin-1 receptor antagonist (IL-1Ra) (Anderson and Mosser, 2002; Mosser and Edwards, 2008; Martinez and Gordon, 2014) (**Figure 1**). Therefore, M2 macrophages play a critical role in the resolution of inflammation and the promotion of tissue repair (Gordon and Martinez, 2010; O’Neill and Pearce, 2016).

## Glycolysis

**Figure 2:**
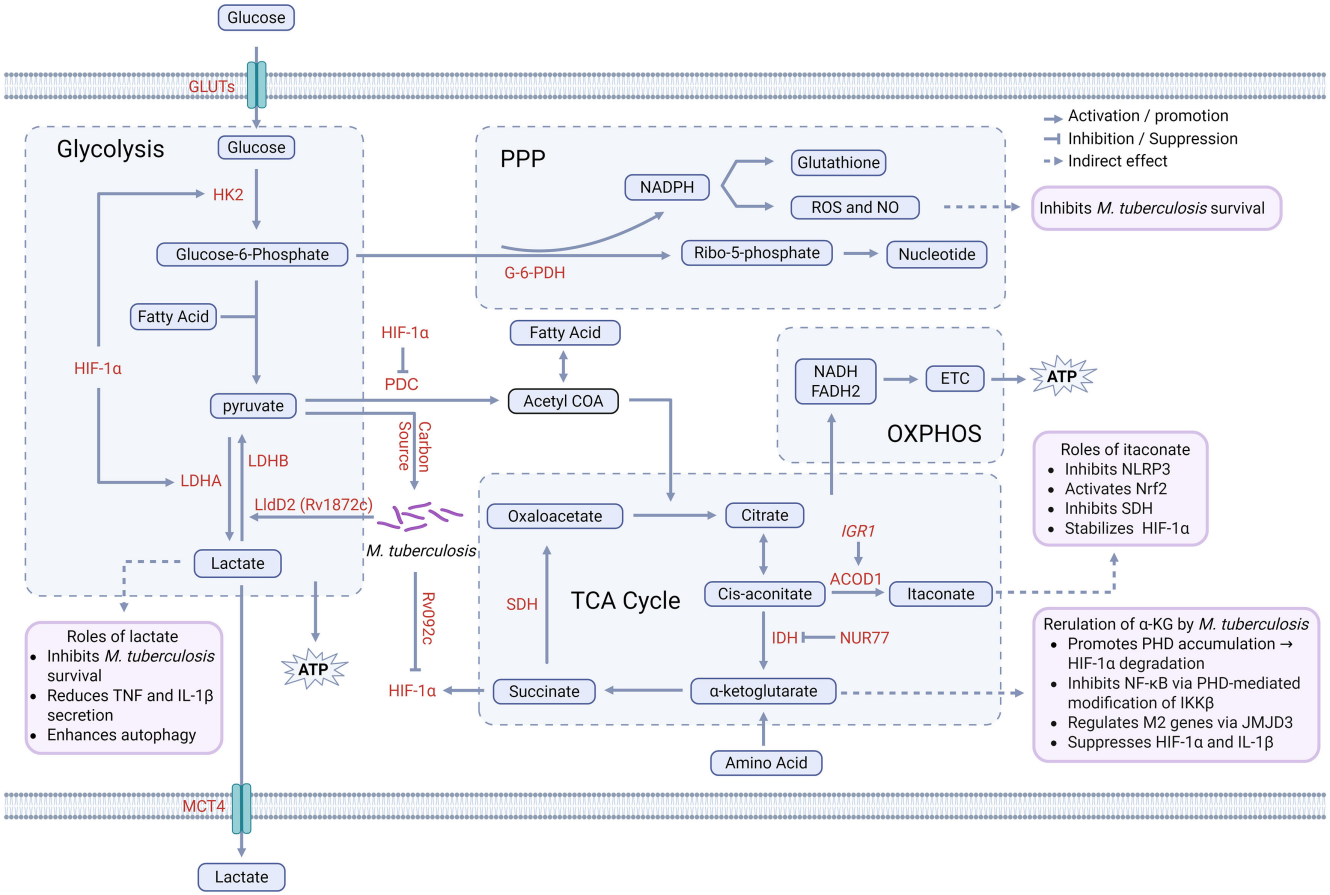
Metabolic pathways involved in macrophage responses during *M. tuberculosis* infection. *M. tuberculosis* infection induces metabolic reprogramming in macrophages, including glycolysis, PPP, and TCA cycle. Increased glycolysis promotes lactate production, while PPP generates NADPH and antimicrobial molecules such as ROS and NO. TCA cycle intermediates, including succinate, itaconate, and α-KG, regulate inflammatory responses, macrophage polarization, and host defense against *M. tuberculosis*. OXPHOS contributes to ATP production through the ETC. *M. tuberculosis* modulates macrophage metabolic states to influence host immune responses and disease progression.

Glycolysis is a fundamental metabolic pathway in which cells internalize extracellular glucose and convert it into pyruvate, lactate, and other metabolites, resulting in the production of a modest amount of ATP and providing intermediates essential for the biosynthesis of nucleic acids, fatty acids, and amino acids (Loftus and Finlay, 2016) (**Figure 2**).

The initial phase of glycolysis is facilitated by glucose transporters (GLUTs) located on the cell membrane, which mediate the import of glucose. Mycobacterial infection enhances glucose uptake in macrophages. *In vitro* studies have shown that *M. tuberculosis* infection upregulates the expression of GLUT1 in murine bone marrow-derived macrophages (BMDM) (Freemerman et al., 2014). Furthermore, animal studies have demonstrated pronounced induction of GLUT6 expression in the lungs of *M. tuberculosis*-infected mice (Shi et al., 2015). These findings indicate that mycobacterial infections promote glucose uptake in macrophages by upregulating glucose transporter expression, thereby supplying substrates for glycolysis.

Hexokinase (HK), the initial rate-limiting enzyme in glycolysis, facilitates the irreversible phosphorylation of glucose to form glucose-6-phosphate. Of the four isoforms (HK1-4), HK2 is the predominant regulator of cellular metabolism (Wilson, 2003). The expression of HK2 is markedly upregulated in almost all cancer cells (Patra et al., 2013; Bao et al., 2018). Mycobacterial infections similarly enhance HK2 expression in macrophages, thus promoting glucose conversion and utilization (Lachmandas et al., 2016). Shi et al. demonstrated that HK2 expression is upregulated in *M. tuberculosis*-infected macrophages, and this regulation is mediated by HIF-1α (Shi et al., 2019). Paradoxically, other findings show that 2-deoxyglucose promotes *M. tuberculosis* growth in BMDM (Hackett et al., 2020). These observations suggest context-dependent heterogeneity in HK2-mediated glycolysis and its impact on host control of infection, highlighting the need for further mechanistic studies.

The product of HK, glucose-6-phosphate, functions as a critical intermediate in glycolysis and serves as an essential substrate for the pentose phosphate pathway (PPP). Glucose-6-phosphate dehydrogenase, the rate-limiting enzyme of the PPP, catalyzes the conversion of glucose-6-phosphate into nicotinamide adenine dinucleotide phosphate (NADPH) and ribose-5-phosphate. These products are essential for nucleotide synthesis and play a role in inflammatory responses within macrophages (Van den Bossche et al., 2017). An upregulation of PPP activity and an increase in NADPH production are characteristic of M1 macrophages. NADPH serves dual roles: it acts as an electron donor for the synthesis of NO and is utilized by NADPH oxidase to produce ROS. Both NO and ROS are directly involved in the macrophage-mediated eradication of pathogens such as *Mycobacterium* species (Chan et al., 1992; Aktan, 2004; Lambeth, 2004; Galván-Peña and O’Neill, 2014). Additionally, NADPH generated via the PPP is crucial for maintaining the reduced state of the antioxidant glutathione, thereby mitigating excessive oxidative stress.

Lactate dehydrogenase (LDH) catalyzes the interconversion of pyruvate and lactate during glycolysis. Specifically, LDHA converts pyruvate to lactate, whereas LDHB catalyzes the reverse reaction. Traditionally, lactate, the end product of glycolysis, was considered a metabolic waste product. However, accumulating evidence highlights its significant pathological roles. In tumor microenvironments, lactate induces macrophage secretion of pro-angiogenic factors, thereby enhancing tumor metastasis and invasion and contributing to poor prognosis (Kiran and Basaraba, 2021). During *M. tuberculosis* infection, HIF-1α upregulates LDHA expression in RAW264.7 macrophages, promoting lactate production. Notably, LDHA-deficient RAW264.7 macrophages show a reduced ability to restrict *M. tuberculosis* growth, suggesting that lactate partially inhibits the intracellular bacterial survival (Rademakers et al., 2011; Osada-Oka et al., 2019). Furthermore, HIF-1α-mediated phosphorylation of the pyruvate dehydrogenase complex (PDC) inhibits the conversion of pyruvate to acetyl-coenzyme A (acetyl-CoA), thereby redirecting metabolic flux from oxidative phosphorylation (OXPHOS) toward glycolysis (Stacpoole, 2017). *M. tuberculosis* genome encodes two quinone-dependent L-lactate dehydrogenase genes, LldD1 (Rv0694) and LldD2 (Rv1872c), which catalyze the irreversible oxidation of lactate to pyruvate (Cole et al., 1998). Notably, the oxidation of lactate mediated by LLDD2 results in the production of pyruvate, providing a carbon source that facilitates *M. tuberculosis* persistence within human monocyte-derived macrophages (Billig et al., 2017). Following infection, macrophages upregulate lactate dehydrogenase A (LDHA) and monocarboxylate transporter 4 (MCT4) to mitigate glycolysis-induced acidosis, suggesting that increased lactate efflux is a crucial host response to *M. tuberculosis* infection (Shi et al., 2015). Furthermore, lactate reduces the secretion of tumor necrosis factor and IL-1β by human macrophages in response to *M. tuberculosis*, while enhancing autophagy-mediated bacterial clearance (Ó Maoldomhnaigh et al., 2021). Apart from Rv1872c, *M. tuberculosis* protein Rv0927c suppresses the activation of the HIF-1α pathway via VHL-mediated ubiquitination and the NF-κB/COX-2 axis, thereby promoting the intracellular survival of *M. tuberculosis* (Xia et al., 2024). Inhibition of HIF-1α, a pivotal transcription factor governing glycolysis, directly impairs the glycolytic capacity of macrophages (Xia et al., 2024). Collectively, these findings indicate that mycobacterial infection influences lactate production through the regulation of LDHA, thereby modulating macrophage immune responses via complex mechanisms to manage bacterial load. Nonetheless, the intricate interaction between macrophages and mycobacteria gives lactate a dual role. Given the limited mechanistic insights into lactate’s role in *M. tuberculosis* infection and disease progression, further investigations are warranted to elucidate how glycolysis-derived lactate shapes macrophage immunomodulatory functions.

## TCA cycle

The TCA cycle, also recognized as the citric acid cycle or Krebs cycle, constitutes a central metabolic hub within mitochondria that integrates carbohydrate, fatty acid, and amino acid metabolism (**Figure 2**). This cyclic pathway facilitates substance exchange and energy transduction essential for most biological processes. Pyruvate derived from glycolysis enters mitochondria, undergoes oxidative decarboxylation via the PDC to form acetyl-CoA, and condenses with oxaloacetate to generate citrate, initiating TCA cycle progression. Glycerol-3-phosphate, derived from glycerol released during fatty acid metabolism, enters the TCA cycle after conversion to glycolytic intermediates. Amino acids contribute directly to the cycle through transamination and deamination reactions, yielding α-ketoglutarate (α-KG) as a key intermediate. The redox coenzymes NADH and FADH_2_, generated via the TCA cycle, act as electron donors for the ETC to produce ATP through OXPHOS (Lin and Duann, 2020). ATP hydrolysis then supplies energy for cellular processes including glycogen synthesis, fatty acid synthesis, and protein synthesis.

Mycobacterial infection modulates the TCA cycle in macrophages by altering the expression or activity of critical enzymes, including isocitrate dehydrogenase (IDH), aconitate decarboxylase 1 (ACOD1), and succinate dehydrogenase (SDH). During the initial phase of *M. tuberculosis* infection in macrophages, the nuclear receptor subfamily 4 group A member 1 (NUR77), whose expression is upregulated by the infection, directly binds to the promoter region of IDH, thereby repressing its expression. This repression facilitates macrophage polarization from M1 to M2 phenotype (Birari et al., 2023). The consequent reduction in IDH expression and activity results in the accumulation of citrate, which is subsequently metabolized by ACOD1 to produce itaconate (Tannahill et al., 2013). Additionally, NUR77 can inhibit the production of NO and IL-1β in an SDH-dependent manner, thereby enhancing *M. tuberculosis* survival within macrophages (Birari et al., 2023).

In mammals, ACOD1 (encoded by *Irg1*) catalyzes cis-aconitate decarboxylation to generate itaconate. *M. tuberculosis* infection markedly upregulates *Irg1* expression (Michelucci et al., 2013; Tannahill et al., 2013; Bomfim et al., 2022). Moreover, *Irg1−/−* mice show increased susceptibility to *M. tuberculosis* compared to wild-type mice, with heightened neutrophil infiltration and aggravated pulmonary damage (Michelucci et al., 2013; Nair et al., 2018; Bomfim et al., 2022). Itaconate exerts extensive immunomodulatory and anti-inflammatory effects. Activation of the NOD-like receptor family pyrin domain containing 3 (NLRP3) inflammasome induces the release of pro-inflammatory cytokines such as IL-1β and interleukin-18, thereby exacerbating inflammatory responses (Ma et al., 2022). However, itaconate inhibits NLRP3 inflammasome activation and consequently reduces the production of pro-inflammatory cytokines (Lin et al., 2022; Ma et al., 2022; Xie et al., 2023; Li et al., 2024; Zhao et al., 2024). Itaconate and its derivative 4-octyl itaconate can promote the nuclear translocation of nuclear factor erythroid 2-related factor 2 (Nrf2) and activate the expression of its downstream antioxidant stress-responsive genes via alkylating Kelch-like ECH-associated protein 1 (Keap1), a negative regulator of Nrf2, thus alleviating tissue damage caused by inflammation (Song et al., 2020; Liu G. et al., 2021). Itaconate can also alkylate key proteins in the nuclear factor-kappa B (NF-κB) signaling pathway, such as IκB kinase β (IKKβ), thereby inhibiting the activation and nuclear translocation of NF-κB and ultimately decreasing the expression of pro-inflammatory cytokines (Ma et al., 2022; Li et al., 2023). Itaconate acts as a competitive inhibitor of SDH. By inhibiting SDH, itaconate reduces ROS generation, thereby suppressing inflammatory mediator release (Anisov et al., 2023; Cordes and Hiller, 2024). However, by inhibiting SDH, itaconate induces succinate accumulation and HIF-1α stabilization, which promotes IL-1β expression and enhances macrophage immune responses against *M. tuberculosis* (Kumar et al., 2019; Kim et al., 2020). Meanwhile, itaconyl-CoA, a degradation product of itaconic acid, has recently been demonstrated to restrict the growth of *M. tuberculosis* (Ruetz et al., 2019). However, *M. tuberculosis* Rv2498c possesses (S)-citryl-CoA lyase (Ccl) activity, converting itaconic acid to pyruvate and acetyl-CoA, thereby sustaining bacterial persistence and pathogenicity (Wang H. et al., 2019).

The TCA cycle intermediate α-KG also promotes macrophage M2 polarization and sustains the M2 phenotype through multiple pathways. HIF-1α is essential for pro-inflammatory factor production in M1 macrophages, whereas α-KG modulates macrophage polarization by regulating HIF-1α expression and activity. In M2 macrophages, α-KG facilitates prolyl hydroxylase (PHD) accumulation, which hydroxylates HIF-1α, leading to its ubiquitination and proteasomal degradation (Liu et al., 2017). This degradation of HIF-1α inhibits M1 polarization while sustaining the M2 phenotype (Liu S. et al., 2021). α-KG suppresses LPS-induced accumulation of HIF-1α and IL-1β in a dose-dependent manner, whereas succinate enhances HIF-1α stability by inhibiting α-KG (Tannahill et al., 2013; Arts et al., 2016). Additionally, α-KG modulates macrophage polarization through mechanisms independent of HIF-1α. For example, glutaminase (GLS) inhibition by BPTES reduces α-KG production and downregulates M2 polarization-related genes (*Arg1*, *Ym1*, *Chil3*, *Retnla* and *Mrc1*) while promoting pro-inflammatory cytokine production. This effect is mitigated by α-KG supplementation, primarily through interaction with the histone demethylase JMJD3, which regulates M2 polarization-related gene (Liu et al., 2017). Simultaneously, α-KG restricts M1 macrophage activation by disrupting the NF-κB pathway via PHD-mediated post-translational modification of IKKβ (Liu et al., 2017). *M. tuberculosis* sulfatide-1 (SL-1) is classified as a cell wall lipid that suppresses host immune responses (Queiroz and Riley, 2017). Its biosynthesis is associated with IL-10 production (Farnia et al., 2026). Additionally, SL-1 abundance varies among different *M. tuberculosis* strains and correlates with TCA cycle flux and M1/M2 polarization in host macrophages (López-Agudelo et al., 2022). These studies collectively demonstrate that *M. tuberculosis*-induced reprogramming of the macrophage TCA cycle, primarily through modulation of key enzymes like IDH and ACOD1 and metabolites like α-KG, promotes a shift towards M2 polarization via multiple pathways, thereby facilitating its survival within macrophages.

## Amino acid metabolism

**Figure 3:**
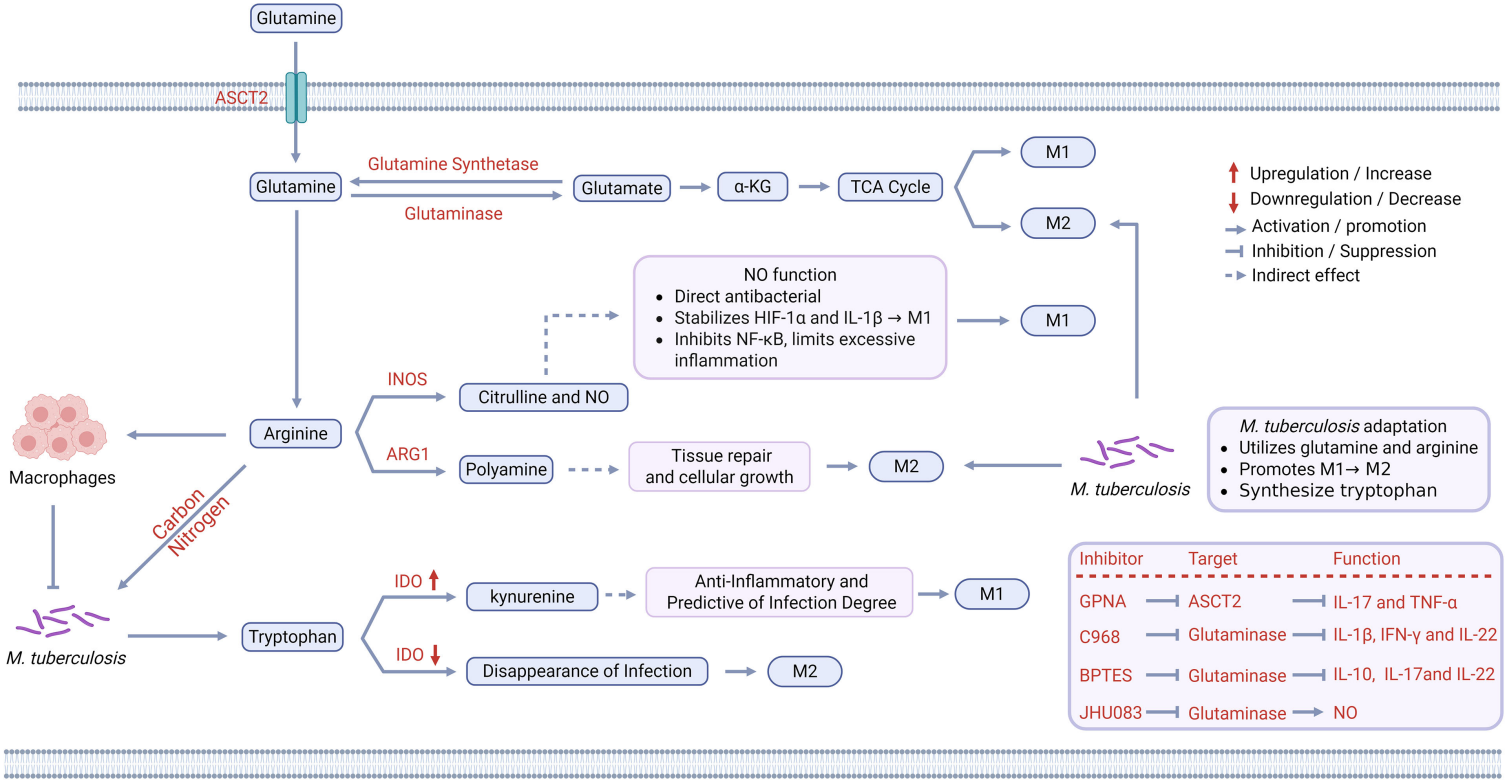
Metabolic crosstalk of glutamine, arginine, and tryptophan in M1/M2 macrophage polarization and functions during *M. tuberculosis* infection. Glutamine is transported into macrophages through ASCT2 and metabolized into glutamate and α-KG, which subsequently enters the TCA cycle to regulate M1/M2 macrophage polarization. Arginine metabolism is mediated through two major pathways: the iNOS pathway, generating citrulline and NO to enhance antibacterial activity and promote M1 polarization, and the ARG1 pathway, producing polyamines involved in tissue repair and M2 polarization. Tryptophan metabolism is regulated by IDO, leading to kynurenine production and modulation of inflammatory responses. In addition, *M. tuberculosis* adapts to the host metabolic environment by utilizing glutamine and arginine, promoting M1-to-M2 differentiation, and synthesizing tryptophan. Potential metabolic inhibitors targeting ASCT2 and glutaminase pathways are also illustrated.

The metabolism of various amino acids, such as arginine, tryptophan, and glutamine, has been documented to undergo modulation during mycobacterial infection (Jiang and Shi, 2021) (**Figure 3**). In *M. tuberculosis*-infected macrophages, arginine has two distinct metabolic pathways: the inducible nitric oxide synthase (iNOS) pathway and the arginase pathway. In the iNOS pathway, nitric oxide synthase catalyzes the decomposition of arginine into citrulline and NO. NO, a reactive nitrogen intermediate with antibacterial activity, inhibits the growth of *M. tuberculosis* by damaging its DNA, proteins, and membrane structures (Rath et al., 2014; Rr and Je, 2021; Zhang K. et al., 2024). In addition, NO stabilizes HIF-1α and IL-1β activity to induce macrophage polarization toward the M1 phenotype, while concurrently inhibiting hyperactive NF-κB signaling to limit excessive inflammation (Braverman and Stanley, 2017; Bailey et al., 2019). In the arginase pathway, arginine is converted by arginase-1 (ARG1) into polyamines, which play crucial roles in cellular growth, development, and tissue repair (Mills et al., 2000). In *M. tuberculosis*-infected macrophages, arginine serves as an essential nutrient that promotes bacterial growth. Studies have shown that *M. tuberculosis* can directly acquire arginine from the host and utilize it as a source of both carbon and nitrogen (Niederweis, 2008; Ogura et al., 2010). *M. tuberculosis* is unable to survive in arginine-depleted culture media, and arginine auxotrophic strains similarly fail to thrive in murine infection models (Gordhan et al., 2002; Mizrahi and Warner, 2018). In contrast, exogenous arginine supplementation enhances macrophage viability and proliferation through NO-independent mechanisms, thereby facilitating pathogen clearance (McKell et al., 2021). These findings indicate that arginine represents a critical nutrient resource for *M. tuberculosis* survival while simultaneously serving as a substrate for macrophage-derived NO production to combat infection. The competitive utilization of arginine thus constitutes a pivotal determinant shaping the outcome of host-pathogen interactions during mycobacterial infection.

Tryptophan, an essential amino acid in humans, is obtained primarily from the diet. Its catabolism proceeds predominantly via the kynurenine pathway, catalyzed by indoleamine 2,3-dioxygenase (IDO), which plays a crucial role in immune regulation. IDO is the rate-limiting enzyme for tryptophan catabolism in extrahepatic tissues, initiating the kynurenine pathway via oxidative cleavage of tryptophan’s indole ring. Under basal conditions, IDO expression is negligible but is markedly upregulated upon inflammation or microbial infection. This induction depletes local tryptophan in the macrophage microenvironment—a change to which many bacteria are highly susceptible. Consequently, IDO was originally characterized as a pivotal effector mechanism mediating host defense against microbial infections (MacKenzie et al., 1998; Däubener and MacKenzie, 1999; MacKenzie et al., 2003). Furthermore, LPS stimulation induces excessive IDO expression in murine lung tissues (Yoshida and Hayaishi, 1978), and significant IDO expression is also etected in macrophages within tuberculous granulomas of non-human primates (Mehra et al., 2013), which further corroborates the close association of IDO with inflammatory and infectious processes. Notably, IDO-mediated reprogramming of tryptophan metabolism is tightly correlated with the progression and prognosis of *M. tuberculosis* infection. On the one hand, the catabolic conversion of tryptophan to kynurenine is markedly enhanced in both latent and active *M. tuberculosis* infection, accompanied by a synchronous elevation in IDO expression levels (Suzuki et al., 2012; Collins et al., 2020; Zhang K. et al., 2024), implying that tryptophan/kynurenine concentrations and IDO expression levels may serve as potential biomarkers for assessing the severity of *M. tuberculosis* infection, while reduced IDO activity could act as a prognostic indicator for the disappearance of infection. On the other hand, systems biology studies have confirmed that IDO is one of the hub genes associated with *M. tuberculosis* survival in human alveolar macrophages (Sadee et al., 2023). Further investigations have shown that *M. tuberculosis* exploits IDO-mediated host metabolic remodeling to facilitate its intracellular persistence. The virulent *M. tuberculosis* H37Rv strain specifically induces elevated kynurenine concentrations and upregulates IDO expression in THP-1-derived macrophages, and IDO-mediated tryptophan depletion promotes the polarization of M2 macrophages, thereby creating a permissive microenvironment for intracellular *M. tuberculosis* colonization (Xiao et al., 2022). *M. tuberculosis* possesses the ability to synthesize tryptophan, whereas tryptophan auxotrophic mutants cannot establish infection in a murine macrophage model (Lott, 2020). However, IDO-mediated tryptophan depletion in host macrophages does not significantly inhibit *M. tuberculosis* growth because this pathogen can replenish tryptophan via its biosynthetic pathway. In this context, the impact of IDO activity on *M. tuberculosis* growth and virulence is minimal (Suzuki et al., 2012; Lott, 2020). In summary, IDO-mediated tryptophan depletion has traditionally been regarded as an antimicrobial mechanism, yet *M. tuberculosis* exhibits resistance to the inhibitory effect of tryptophan depletion. This resistance stems from its ability to synthesize tryptophan autonomously and to utilize the tryptophan metabolite kynurenine to promote M1-to-M2 macrophage polarization.

Glutamine metabolism critically shapes macrophage polarization and their capacity to combat *M. tuberculosis* (Jiang et al., 2022; Parveen et al., 2023; Yu et al., 2024). During *M. tuberculosis* infection, glutamine metabolism provides major carbon and nitrogen sources and drives macrophage metabolic reprogramming (Collins et al., 2020; McKell et al., 2021; Sadee et al., 2023). This reprogramming elicits pro-inflammatory and antimicrobial responses similar to those in LPS/IFN-γ-activated M1 macrophages (Saha et al., 2017; Jiang et al., 2022). Conversely, treatment with glutaminase (GLS) inhibitors, such as BPTES and CB-839, impairs M1 polarization and promotes an M2 phenotype, thereby enhancing intracellular *M. tuberculosis* growth (Jiang et al., 2022). Transcriptomic analyses of *M. tuberculosis*-infected human peripheral blood mononuclear cells and blood samples from patients with tuberculosis demonstrated the upregulation of genes associated with glutamine transport and utilization, including glutamate-ammonia ligase, glutamate oxaloacetate transaminase 1 and solute carrier family 1 member 5 (SLC1A5). This upregulation coincided with increased glutamine-derived metabolites, including glutamate and α-KG, which fuel the TCA cycle. Furthermore, inhibiting the ASCT2 transporter (encoded by SLC1A5) with GPNA reduces tumor necrosis factor-α (TNF-α) and interleukin-17 (IL-17) production in macrophages. Inhibiting the GLS with BPTES suppresses the production of interleukin-10, interleukin-17 and interleukin-22, whereas C968 inhibits the release of IL-1β, IFN-γ and interleukin-22 (Koeken et al., 2019). Wells et al. reported that glutamine-overloaded murine macrophages show elevated IL-1β, tumor necrosis factor-α (TNF-α), and interleukin-6 (IL-6) upon LPS stimulation (Wells et al., 1999). Additionally, glutamine is indispensable for NO production. In LPS-stimulated macrophages, glutamine-derived arginine via the urea cycle serves as an iNOS substrate, promoting NO generation (Murphy and Newsholme, 1998; Bellows and Jaffe, 1999). Interestingly, integrated metabolomic and transcriptomic studies identify glutamine metabolism as a hallmark of M2 macrophages, contributing one-third of carbon to TCA cycle metabolites versus only one-fifth in M1 macrophages. Glutamine deprivation impairs M2 macrophages but exerts minimal effects on M1 macrophages (Jha et al., 2015). Moreover, inhibition of glutamine synthetase activity in M2 macrophages shifts polarization toward HIF-1α-mediated M1 macrophages (Palmieri et al., 2017). A recent study highlighted the differential regulation of glutamine metabolism and GLS expression by mycobacteria of distinct virulence. *M. tuberculosis* infection inhibits macrophage autophagy, decreases glutamine levels and downregulates GLS expression, whereas BCG infection promotes autophagy, increases glutamine content and enhances GLS expression (Yu et al., 2024). Glutamine also acts as a precursor for the synthesis of multiple macromolecules, such as proteins and nucleotides, in *M. tuberculosis* (Guida et al., 2024). JHU083, a novel anticancer agent targeting glutamine metabolism and T-cell stimulation, inhibits *M. tuberculosis* proliferation both *in vitro* and *in vivo* (Leone et al., 2019; Parveen et al., 2023). JHU083 suppresses glutamine metabolism to directly or indirectly affect *M. tuberculosis* metabolic pathways essential for survival; for instance, the methionine biosynthesis pathway of *M. tuberculosis* is critical for its intracellular survival in the host (Nain et al., 2025). Inhibition of glutamine metabolism may interfere with the metabolic adaptability and replication of *M. tuberculosis* by modulating amino acid availability (Guida et al., 2024; Rehman et al., 2024). Macrophages treated with JHU083 also exhibit increased NO production and enhanced antimicrobial activity. Nevertheless, JHU083 loses its therapeutic efficacy in a murine model of *M. tuberculosis* infection with immunodeficiency (Parveen et al., 2023).Together, these findings reveal the complex regulation of glutamine metabolism in macrophage function during *M. tuberculosis* infection. Its catabolic products-including TCA cycle intermediates and NO-support the M1 phenotype. However, excessive activation or specific pharmacological inhibition, for example by using BPTES or CB-839, suppresses M1 polarization and shifts the balance toward M2 polarization, ultimately promoting bacterial survival. Notably, divergent modulation of this pathway by *M. tuberculosis* versus BCG, along with context-dependent effects of inhibitors such as JHU083, underscores the complexity of host-pathogen metabolic interplay. This complexity necessitates careful consideration in therapeutic strategies.

## Fatty acid metabolism

Fatty acid oxidation (FAO) in macrophages is a multi-step process consisting of three core sequential events, including fatty acid activation, translocation, and mitochondrial β-oxidation. Carnitine palmitoyl transferases (CPT1 and CPT2) are the rate-limiting enzymes of the FAO pathway. M2 macrophage polarization is tightly correlated with FAO. These macrophages predominantly rely on OXPHOS to fulfill their energy requirements, whereas FAO sustains OXPHOS by supplying acetyl-CoA to fuel the TCA cycle (Huang et al., 2014; Batista-Gonzalez et al., 2019). FAO inhibition modulates the intracellular survival of mycobacteria. For instance, genetic ablation of genes involved in fatty acid uptake and β-oxidation triggers AMPK activation, HIF-1α stabilization, and autophagy initiation. Together, these alterations reduce intracellular *M. tuberculosis* survival (Simwela et al., 2025). Separately, microRNA-33 blocks macrophage FAO by transcriptionally suppressing CPT1, thereby inhibiting intracellular *M. tuberculosis* proliferation (Chandra et al., 2020). The underlying mechanism may involve the rapid production of mitochondrial-derived ROS following FAO inhibition, which further facilitates NADPH oxidase recruitment and autophagy induction, and this signaling cascade ultimately restricts *M. tuberculosis* growth (Chandra et al., 2020). Additionally, trimetazidine, a small-molecule FAO inhibitor, reduces pathogen burden in *M. tuberculosis*-infected murine models (Chandra et al., 2020). Nevertheless, conflicting findings suggest that macrophage FAO exerts anti-pathogenic effects by enhancing ROS generation (Hall et al., 2013; Batista-Gonzalez et al., 2019). ROS are direct mediators of the microbicidal activity in phagocytes (Hall et al., 2013; Tur et al., 2020). Notably, IRG1 encodes a mitochondrial enzyme that regulates ROS production during FAO-fueled OXPHOS, thereby contributing to pathogen clearance (Hall et al., 2013). Beyond modulating pathogen survival, FAO also participates in *M. tuberculosis*-induced inflammatory responses. In BCG-induced inflammation, BCG upregulates dual-specificity phosphatase 5 (DUSP5) expression via the TLR2-MAPKs pathway, which subsequently promotes FAO activity (Luo et al., 2024). Further mechanistic studies showed that DUSP5 silencing alleviates lung tissue damage, reduces pro-inflammatory cytokine expression, and suppresses NF-κB activation. These observations indicate that DUSP5-mediated FAO exerts a pro-inflammatory role in *M. tuberculosis*-induced inflammatory responses (Luo et al., 2024). In summary, the role of FAO in macrophage polarization and antimicrobial responses remains controversial. The duality of β-oxidation in promoting either anti-inflammatory (M2 macrophages) or pro-inflammatory (M1 macrophages) responses underscores its context-dependent role in host-pathogen interactions.

Acetyl-CoA, produced through various metabolic pathways, serves as the substrate for fatty acid synthesis. Fatty acids are indispensable for cell growth and proliferation and provide precursors for other intracellular metabolic pathways. Fatty acid synthesis (FAS) is essential for M1 macrophages to perform their pro-inflammatory functions. The mammalian target of rapamycin participates in the *de novo* lipogenesis pathway via the transcription factor sterol regulatory element-binding protein (SREBP), which regulates the expression of genes involved in fatty acid and cholesterol biosynthesis (Im et al., 2011; Singh and Subbian, 2018). The SREBP isoform, SREBP-1a, is markedly upregulated in LPS-induced M1 macrophages. LPS induces fatty acid and Nlrp1a production in response to fatty acid depletion during M1 macrophage proliferation by enhancing NF-κB/Sp1 co-activation of the SREBP-1a promoter. Nlrp1a is an inflammasome component that activates Caspase-1 to cleave IL-1β during pro-inflammatory responses. Consequently, SREBP-1a-deficient murine macrophages exhibit reduced IL-1β production (Im et al., 2011; Singh and Subbian, 2018). Another key enzyme of fatty acid synthesis is fatty acid synthase (FASN), which is required for LPS-induced M1 macrophage activation. Studies demonstrate that FASN inhibitors prevent IκB degradation, significantly reduce the phosphorylation of p65, IKKα/β, and TAK1, and concurrently block IL-1β production (Carroll et al., 2018). Together, these observations highlight the indispensable role of fatty acid synthesis in M1 pro-inflammatory responses, intricately regulated by SREBP-1a and FASN. This regulation establishes a connection between fatty acid metabolism, inflammatory signaling via NF-κB, and inflammasome activation, specifically IL-1β generation. Furthermore, triglycerides and cholesterol esters derived from exogenous fatty acids accumulate significantly in M1 macrophages (Morgan et al., 2021). The *de novo* synthesis of saturated fatty acids (SFA) is governed by the rate-limiting enzyme FASN, while monounsaturated fatty acids (MUFA) are synthesized from SFA by the rate-limiting enzyme stearoyl-CoA desaturase (Guillou et al., 2010; Laval et al., 2021b). The upregulation of SFA and MUFA mRNA expression is correlated with FASN transcription (Laval et al., 2021b). Following *M. tuberculosis* and BCG infection, there is an observed increase in the oleic acid to stearic acid ratio during the conversion of SFA to MUFA. This finding further substantiates the critical role of fatty acid synthesis in the context of mycobacterial infection in BMDM (Laval et al., 2021b). Specifically, *M. tuberculosis* infection enhances SFA and MUFA biosynthesis in BMDM via TLR2/4 activation (Laval et al., 2021b). Collectively, these findings highlight that mycobacterial infection reprograms fatty acid metabolism in macrophages, where the dynamic balance between fatty acid oxidation and synthesis critically regulates macrophage polarization, inflammatory responses, and antimicrobial functions, albeit in a complex and context-dependent manner that influences both host defense and immunopathology.

Upon *M. tuberculosis* infection of macrophages, reprogramming of lipid metabolism drives extensive lipid droplet (LD) formation. This process serves as a critical event in macrophages and *M. tuberculosis* interactions and exerts multifaceted effects on *M. tuberculosis* infection, intracellular survival and latent persistence (Agarwal et al., 2021; de Almeida et al., 2023; Hüsler et al., 2023; Kim and Shin, 2023). Following infection with *M. tuberculosis*, macrophages enhance the uptake of extracellular lipids and facilitate LD biogenesis by remodeling intracellular lipid metabolic pathways (Agarwal et al., 2021; Laval et al., 2021a). Mannose-capped lipoarabinomannan (ManLAM), a key lipoglycan of *M. tuberculosis*, induces LD accumulation in macrophages (Nag et al., 2026). Moreover, heat-killed *M. tuberculosis* (HKMT) activates the lipid scavenger receptor CD36, thereby promoting intracellular lipid deposition in macrophages (AlSaeed et al., 2024). During *M. tuberculosis* infection, lipid metabolism pathways, particularly those regulated by peroxisome proliferator-activated receptors (PPARs), exert essential functions in LD formation (Tanigawa et al., 2021). Among these, PPARγ acts as a core transcription factor controlling lipid metabolism, and its activation facilitates LD accumulation (Tanigawa et al., 2021; AlSaeed et al., 2024). *M. tuberculosis* utilizes macrophage-derived fatty acids as carbon sources to sustain its intracellular infection (Laval et al., 2021a; Kim and Shin, 2023; Simwela et al., 2025). Upregulated expression of the LD surface protein Perilipin 2 (PLIN2) is associated with the survival and replication of intracellular *M. tuberculosis* (Zhang X. et al., 2024). *M. tuberculosis* phthiocerol dimycocerosate inhibits NADPH oxidase and autophagy, thereby impairing the bactericidal capacity of macrophages (E and Ja, 2025). In addition, *M. tuberculosis* disrupts lysosomal function to induce lipid storage within phagolysosomes, ultimately driving foam cell formation, facilitating tissue destruction and sustaining persistent bacterial infection (Rombouts and Neyrolles, 2023). LDs can further recruit and compartmentalize key enzymes involved in eicosanoid synthesis, including cytosolic phospholipase A2 (cPLA2), cyclooxygenase (COX), and lipoxygenase (LOX), thereby modulating the production of inflammatory mediators (de Almeida et al., 2023). *M. tuberculosis* infection-induced LD accumulation is closely linked to the recruitment of anti-inflammatory cytokines such as TGF-β and IL-10. These cytokines contribute to the formation of an immune-tolerant microenvironment and promote polarization of macrophages toward an M2 phenotype, which ultimately favors intracellular bacterial survival (Agarwal et al., 2021). Therefore, *M. tuberculosis*-induced LD formation is not merely a storage phenomenon but a central immunometabolic event. By compartmentalizing eicosanoid synthesis machinery and fostering an M2-like cytokine milieu (TGF-β, IL-10), LDs actively contribute to resolving inflammation and creating an immune-privileged niche. This positions LDs as critical structural hubs that integrate fatty acid metabolism with the suppression of anti-mycobacterial immunity, thereby promoting bacterial persistence.

## The biphasic metabolic dynamics of macrophage responses to *M. tuberculosis* infection

*M. tuberculosis* infection in macrophages triggers a host immunometabolic response that is not a single static process but rather exhibits time-dependent biphasic dynamics: an early pro-inflammatory glycolysis-dominant phase followed by a late anti-inflammatory phase characterized by oxidative phosphorylation and fatty acid oxidation (Shi et al., 2019). These biphasic dynamics represent both a host immune strategy to eliminate the pathogen and an evolutionary product by which *M. tuberculosis* actively hijacks host metabolism to establish a persistence niche.

During the early phase of infection, macrophages rapidly polarize toward an M1 phenotype, with a core metabolic hallmark being the shift from OXPHOS to aerobic glycolysis (the Warburg effect) (Shi et al., 2016; Van den Bossche et al., 2017). Upon recognition of *M. tuberculosis* by TLR2/4, downstream NF-κB signaling activates HIF-1α, which acts as a “master metabolic switch” to directly upregulate the transcription of rate-limiting glycolytic enzymes (hexokinase, phosphofructokinase, and pyruvate kinase) (Shi et al., 2019). Simultaneously, “block” at two key nodes of the TCA cycle leads to accumulation of itaconate and succinate (Ryan and O’Neill, 2020). Succinate stabilizes HIF-1α by inhibiting PHD, thereby further amplifying IL-1β secretion (Ryan and O’Neill, 2020). Meanwhile, glutaminolysis replenishes α-KG to sustain pro-inflammatory signaling required for M1 polarization (Jiang et al., 2022). Furthermore, PKM2-mediated glycolysis promotes NLRP3 inflammasome activation (Xie et al., 2016). At this stage, the host combats the bacteria through production of ROS, release of NO, and nutrient restriction strategies such as iron sequestration (Huang et al., 2018).

As infection progresses, the metabolic state of macrophages undergoes a fundamental reversal. Using extracellular flux analysis, Cumming et al. demonstrated that live *M. tuberculosis*-infected human monocyte-derived macrophages exhibit a unique “quiescent energy phenotype,” characterized by markedly reduced glycolytic and TCA cycle fluxes, decreased mitochondrial dependence on glucose, and increased reliance on exogenous fatty acids (Cumming et al., 2018). This transition is closely associated with the formation of foamy macrophages, in which extensive LD accumulation not only provides fatty acids as a carbon source for the bacteria but also serves as a platform for eicosanoid synthesis, thereby biasing toward the PGE_2_-mediated anti-inflammatory pathway (Genoula et al., 2020; Shim et al., 2020b). In parallel, OXPHOS sustained by FAO supports the secretion of anti-inflammatory cytokines such as IL-10 and TGF-β (Park et al., 2021). The *M. tuberculosis* virulence factor ESAT-6 has been shown to first drive M1 polarization and subsequently promote a switch to the M2 phenotype (Refai et al., 2018). Moreover, *M. tuberculosis* suppresses host glycolysis and IL-1β production by upregulating miR-21, which restricts PFK-M expression (phosphofructokinase, muscle isoform) (Hackett et al., 2020).

However, it should be noted that the biphasic dynamics exhibit heterogeneity: the metabolic impairment pattern of human alveolar macrophages following *M. tuberculosis* infection is not entirely consistent with that of bone marrow-derived macrophages *in vitro*, and type I interferon signaling can independently reduce the overall energy metabolism of macrophages (Olson et al., 2021; Mendonca et al., 2022). Furthermore, the diversity of *M. tuberculosis* strains influences both the extent of host metabolic reprogramming and the pattern of inflammasome activation, suggesting that biphasic dynamics may exhibit significant variation across different clinical contexts (Fernandes et al., 2025).

## Perspectives

It is well-established that intracellular metabolic pathways are intricately interconnected. Glucose-6-phosphate generated through glycolysis participates in the PPP. Acetyl-CoA derived from glycolysis and fatty acid oxidation feeds into the TCA cycle. Similarly, glucogenic amino acids contribute to glucose synthesis via gluconeogenesis. Furthermore, the labyrinthine network of intracellular signaling pathways integrates metabolic activity with immune cell processes such as proliferation, activation, differentiation, and bactericidal functions, thereby modulating localized or systemic immune responses. For instance, glycolysis provides rapid energy production to support immune cell proliferation, while fatty acid oxidation and the TCA cycle generate larger quantities of energy over extended periods, facilitating tissue repair. The PPP supplies nucleic acid precursors for immune cell expansion, lipid biosynthesis provides structural components for cellular membranes, and amino acid metabolites like ROS exert direct antimicrobial effects.

Immunometabolic reprogramming has emerged as a crucial interdisciplinary field that bridges immunology and metabolism. Research into the metabolic reprogramming of immune cells under inflammatory conditions, including infections such as *M. tuberculosis*, has significantly enhanced our understanding of disease pathogenesis and progression. Consequently, the host’s response to *M. tuberculosis* and similar inflammatory conditions is influenced by the dynamic interplay between the metabolic strategies of the pathogen and the metabolic states of the host’s immune cells. Nevertheless, further research is necessary to elucidate the complex interactions among metabolic pathways and to guide immune cell differentiation towards outcomes that are favorable to the host. Simultaneously, comprehensive mechanistic investigations into the ways in which *M. tuberculosis* manipulates these pathways are crucial for reducing bacterial evasion of the immune system within host cells. The metabolic pathways and their associated metabolites in immune cells are not only vital for maintaining cellular homeostasis but also play a significant role in regulating immune responses. Although translating immunometabolic insights into clinical applications remains challenging, the growing understanding of mycobacterial pathogenesis highlights metabolic pathways as promising therapeutic targets. Modulating the metabolic states of immune cells could represent an innovative approach to managing mycobacterial infection, presenting new opportunities to disrupt the survival strategies of pathogens and enhance host defense mechanisms.
